# Baseline and early digital [^18^F]FDG PET/CT and multiparametric MRI contain promising features to predict response to neoadjuvant therapy in locally advanced rectal cancer patients: a pilot study

**DOI:** 10.1097/MNM.0000000000001703

**Published:** 2023-05-03

**Authors:** Floris A. Vuijk, Shirin Feshtali Shahbazi, Wyanne A. Noortman, Floris H.P. van Velden, Petra Dibbets-Schneider, Andreas W.K.S. Marinelli, Peter A. Neijenhuis, Roderick Schmitz, Eidrees Ghariq, Laura A. Velema, Femke P. Peters, Frits Smit, Koen C.M.J. Peeters, Sofieke J.D. Temmink, Stijn A.L.P. Crobach, Hein Putter, Alexander L. Vahrmeijer, Denise E. Hilling, Lioe-Fee de Geus-Oei

**Affiliations:** a Department of Surgery, Leiden University Medical Center; b Department of Radiology, Leiden University Medical Center, Leiden; c Department of Radiology, Section of Nuclear Medicine, Leiden University Medical Center; d Biomedical Photonic Imaging Group, University of Twente, Enschede; e Department of Surgery, Haaglanden Medisch Centrum, Den Haag; f Department of Surgery, Alrijne Ziekenhuis, Leiderdorp; g Department of Surgery, Groene Hart Ziekenhuis, Gouda; h Department of Radiation Oncology, Leiden University Medical Center; i Department of Radiation Oncology, Antoni van Leeuwenhoek Hospital, Amsterdam; j Department of Pathology, Leiden University Medical Center; k Department of Medical Statistics, Leiden University Medical Center, Leiden; l Department of Surgical Oncology and Gastrointestinal Surgery, Erasmus MC Cancer Institute, University Medical Center Rotterdam; m Department of Surgery, Ijsselland Ziekenhuis, Capelle a/d IJssel; n Department of Radiation Science & Technology, Technical University Delft, The Netherlands

**Keywords:** computed tomography, fluorodeoxyglucose, multiparametric resonance imaging, neoadjuvant therapy, PET, rectal neoplasms

## Abstract

In this pilot study, we investigated the feasibility of response prediction using digital [^18^F]FDG PET/computed tomography (CT) and multiparametric MRI before, during, and after neoadjuvant chemoradiation therapy in locally advanced rectal cancer (LARC) patients and aimed to select the most promising imaging modalities and timepoints for further investigation in a larger trial.

**Methods:**

Rectal cancer patients scheduled to undergo neoadjuvant chemoradiation therapy were prospectively included in this trial, and underwent multiparametric MRI and [^18^F]FDG PET/CT before, 2 weeks into, and 6–8 weeks after chemoradiation therapy. Two groups were created based on pathological tumor regression grade, that is, good responders (TRG1-2) and poor responders (TRG3-5). Using binary logistic regression analysis with a cutoff value of *P* ≤ 0.2, promising predictive features for response were selected.

**Results:**

Nineteen patients were included. Of these, 5 were good responders, and 14 were poor responders. Patient characteristics of these groups were similar at baseline. Fifty-seven features were extracted, of which 13 were found to be promising predictors of response. Baseline [T2: volume, diffusion-weighted imaging (DWI): apparent diffusion coefficient (ADC) mean, DWI: difference entropy], early response (T2: volume change, DWI: ADC mean change) and end-of-treatment presurgical evaluation MRI (T2: gray level nonuniformity, DWI: inverse difference normalized, DWI: gray level nonuniformity normalized), as well as baseline (metabolic tumor volume, total lesion glycolysis) and early response PET/CT (Δ maximum standardized uptake value, Δ peak standardized uptake value corrected for lean body mass), were promising features.

**Conclusion:**

Both multiparametric MRI and [^18^F]FDG PET/CT contain promising imaging features to predict response to neoadjuvant chemoradiotherapy in LARC patients. A future larger trial should investigate baseline, early response, and end-of-treatment presurgical evaluation MRI and baseline and early response PET/CT.

## Introduction

Patients diagnosed with locally advanced rectal cancer (LARC) are currently treated with neoadjuvant chemoradiotherapy (nCRT), prior to surgical resection. The goal of nCRT is to downsize and downstage rectal cancer, thereby improving the rate of complete resections and lowering the risk of local recurrence [1]. The majority of patients have a partial tumor response after nCRT [1], while in 15–20% this even results in a pathological complete response (pCR) of all tumor tissue [1,2]. Most recently, results from the RAPIDO trial demonstrate even higher rates of pCR (28%) after neoadjuvant short-course radiotherapy followed by chemotherapy [3]. Unfortunately, not all patients respond well to nCRT, but the exact number of nonresponders is uncertain [4].

According to current guidelines, treatment stratification and response assessment are performed using MRI and in selected cases, rectoscopy [5]. MRI features include the tumor-node-metastasis stage, extramural vascular invasion (EMVI), and tumor distance to the mesorectal fascia [6]. Unfortunately, current imaging modalities are unable to predict response to nCRT accurately. In recent years, the watch-and-wait strategy has been implemented for patients with clinical complete response (cCR) after neoadjuvant therapy, with excellent long-term outcomes [1,7]. By means of improved stratification before or early after the onset of nCRT, a precise selection of patients might be possible. In patients predicted to respond well, the (watchful) waiting period before surgery could be prolonged, possibly increasing the rate of cCR. Accurate identification of cCR patients can prevent futile surgery and its associated morbidity and mortality [8]. In patients with a predicted poor response, unbeneficial continuation of nCRT, therapy-related toxicity and unwanted delay in the initiation of a potentially effective treatment could be avoided.

Currently, 2-[^18^F]fluoro-2-deoxy-d-glucose ([^18^F]FDG) PET combined with computed tomography (PET/CT) is advised in the national guideline for the detection of recurrence of rectal cancer in case of increased carcinoembryonic antigen levels [9]. Many MRI and [^18^F]FDG PET/CT features have been investigated separately to predict response to nCRT before or early after the onset of nCRT [10–21]. The combination of both modalities could possibly have complimentary value to predict response. Available data in the literature are insufficient to evaluate this approach, and no studies have investigated the application of digital PET/CT in this field [13,16,19]. Owing to its increased energy resolution and time-of-flight performance, digital PET/CT has the potential to improve the quantification of small or heterogeneous tumors and thereby provide more accurate metabolic information on tumor response, and might (in combination with multiparametric MRI) facilitate improved response prediction to nCRT.

In this pilot study, we investigate the feasibility of response prediction using digital [^18^F]FDG PET/CT and multiparametric MRI before, during, and after nCRT in LARC patients and aim to determine the most promising imaging modalities and time points for further investigation.

## Materials and methods

### Patient population

A multicenter, nonrandomized prospective study was performed in patients admitted to the Leiden University Medical Center (*n* = 8), Haaglanden Medical Center (*n* = 6), Alrijne Hospital Leiderdorp (*n* = 4), and Groene Hart Hospital (*n* = 1), diagnosed with (biopsy proven) LARC and treated according to national guidelines. Eligible patients were selected at multidisciplinary meetings and asked for participation during their outpatient clinic visits. Treatment consisted of nCRT (25 × 2 Gy combined with 825 mg/m^2^ bid capecitabine 5 days per week), followed by reevaluation after 6–8 weeks. Surgery followed within 4–6 weeks after reevaluation. In case of a near complete response, reevaluation was repeated after 6–8 weeks. In the case of cCR, follow-up was initiated according to the watch-and-wait protocol [7]. The study was conducted in concordance with the Declaration of Helsinki, and was approved by the Leiden-Den Haag-Delft medical ethics review board and the local boards of participating centers. All subjects provided written informed consent. The study was registered in the Netherlands Trial Register (identification number NL-756). Including standard of care imaging (rectoscopy, MRI scan of abdomen, and CT scan of the chest and abdomen), all patients underwent [^18^F]FDG PET/CT and multiparametric MRI before nCRT, 10–14 days after nCRT onset (early response evaluation), and 6–8 weeks after the last treatment (end-of-treatment presurgical evaluation).

### Data acquisition and image reconstruction

All digital [^18^F]FDG PET/CT scans of the lower abdomen were acquired on the same scanner, a Vereos PET/CT (Philips Healthcare, Best, the Netherlands). All acquisitions and reconstructions were in accordance with European Association of Nuclear Medicine (EANM) guidelines for tumor PET imaging version 2.0 [22]. Prior to PET/CT scanning, patients fasted for 6 h and were prehydrated using 1 L of water. [^18^F]FDG was dosed using the quadratic formula: 379 (MBq·min·bed^−1^·kg^−2^) × [patient weight (kg)/75]^2^/emission acquisition duration per bed position (min·bed^−1^) with a factor of 379 MBq·min·bed^−1^·kg^−2^. Patients received 20 mg intravenous furosemide 15 min post-injection. Patients underwent a low-dose CT scan for attenuation correction 60 (55–65) min post-injection (120 kV, 35 mA_eff_), followed by a PET scan of 5 min per bed position. Reconstructed PET images had a voxel size of 4 × 4 × 4 mm. Multiparametric MRI of the lower abdomen was made on various systems, and included T2- and diffusion-weighted imaging (DWI) sequences. Patients underwent bowel preparation using a 5 ml Microlax enema three hours before imaging (Johnson and Johnson, New Brunswick, New Jersey, USA). Further details are described in Supplementary Table 1, Supplemental digital content 1, http://links.lww.com/NMC/A246.

### Quantitative image analysis

MRI assessment was performed by a board-certified abdominal radiologist (S.F.S., 11 years of experience), using Sectra IDS7 software (version 21.2; Sectra AB, Linköping, Sweden). Apparent diffusion coefficient (ADC) values were calculated from the DWI image. Volumes of interest (VOIs) were drawn manually (F.V. under the supervision of S.F.S.) to include the primary tumor on the DWI and T2 maps. Various quantitative features were extracted using 3DSlicer (version 4.11) [23] and PyRadiomics (version 3.0) which was running in Python (version 3.7; Python Software Foundation, Wilmington, Delaware, USA) [24]. First, following the methodology of Schurink *et al.* [19], the following features were extracted from the VOIs: T2 mesh volume, T2 entropy, DWI mesh volume, mean ADC, ADC entropy, and their respective response indices. Second, to allow full comparison to the results from Schurink *et al.* [19,20] and following recent promising results from Delli Pizzi *et al.* [25], 105 radiomic features were extracted from the T2 baseline images for additional radiomic analysis: shape [14], first order [18], gray level cooccurrence matrix [22], gray level run length matrix [16], gray level size zone matrix [16], gray level dependence matrix [14] and neighboring gray-tone difference matrix [5] features. Images were interpolated to isotropic voxels of 2.00 × 2.00 × 2.00 mm^3^ using B-spline interpolation, with grids aligned by the input origin and only covering the VOI. Both T2 and DWI images were normalized to a mean of 300 and a SD of 100, allowing comparison of the relative gray values between patients [26]. Features were extracted using a fixed bin size, which was determined in such a way that most VOIs contained between 30 and 130 bins. This resulted in a bin size of 5 and 15 for T2 and DWI images, respectively.

PET/CT assessment was performed by a board-certified nuclear medicine physician (L.G., 25 years of experience), using Sectra IDS7 software (version 21.2; Sectra AB, Linköping, Sweden). VOIs were automatically delineated with an isocontour threshold of 50% of the maximum standardized uptake value (SUV_max_) using IntelliSpace Portal (version 9.0; Koninklijke Philips N.V., Amsterdam, the Netherlands). The following features were included in the analysis with their corresponding response indices based on the following articles. Joye *et al.* pooled data from 25 studies investigating [^18^F]FDG PET/CT and found the following features to be promising predictors for response [17]: the SUV_max_ post-therapy, response indices of the SUV_max_, the metabolic tumor volume [MTV, obtained using a peak standardized uptake value corrected for lean body mass (SUL_peak_) threshold of 50%] and total lesion glycolysis (TLG, SUV_mean_ × MTV). All features were body weighted, except SUL_peak_, which was weighted using the lean body mass following the methodology described in PERCIST 1.0 and by O *et al* [27]. They advise the use of SUL_peak_ as exploratory data when the liver is not present in all scans. No radiomic feature analysis was performed on data from [^18^F]FDG PET/CT, as this has not been described in literature before.

### Pathology

Pathological assessment of the resection specimen was performed according to the Dutch national guidelines [9]. In addition to this, the extent of tumor regression was evaluated according to Mandard’s tumor regression grade (TRG) by the local board-certified pathologist [28]. Mandard’s TRG classifies response to given therapy into five classes based on the number of vital tumor cells and the extent of therapy-induced fibrosis. When classified TRG 1, no residual tumor cells were seen, and the patient is considered to have a pathologic complete response (pCR). A regrowth-free survival time of >6 months was considered a surrogate endpoint for TRG1 in patients with a cCR in watch-and-wait follow-up.

### Statistical analysis

Statistical analysis was performed using SPSS (version 25; IBM SPSS, Inc., Chicago, Illinois, USA) and R (version 3.6.0; R Foundation for Statistical Computing, Vienna, Austria). For statistical analysis, patients were divided into two groups based on the pathological TRG or regrowth-free follow-up in the case of watch-and-wait: good responders (TRG1-2) and poor responders (TRG 3–5). Descriptive data were displayed as mean ± SD or median (interquartile range), depending on the distribution of data. Non-parametric data were compared using the Mann–Whitney *U* test, whereas parametric data were compared using a *T*-test. Results were considered significant when *P* < 0.05. Promising imaging features were selected using binary logistic regression, after dividing through their respective SD. Due to the small sample size and large amount of tested features, MRI and PET/CT features were considered promising when a *P* value ≤0.2 was reached.

Unsupervised radiomic feature selection using redundancy filtering and factor analysis was performed using FMradio (Factor Modeling for Radiomics Data, package version 1.1.1; Amsterdam UMC, Amsterdam, the Netherlands), developed for R (version 3.6.0; R Foundation for Statistical Computing, Vienna, Austria) [29]. The large feature dimensionality compared to the small sample size might result in overfitting and deteriorates the generalizability of the radiomic model. Therefore, one feature was selected for every 10 subjects [30]. Features were scaled (centered around 0, variance of 1) to avoid the features with the largest value would dominate the analysis. Redundancy filtering on the Pearson correlation matrix was performed with a threshold of *τ* = 0.95 and from each group, one feature was retained. Factor analysis of the redundancy-filtered correlation matrix was performed and two factors (19 patients) were selected per sequence and time point. The sampling adequacy of the model was determined by the Kaiser–Meier–Olkin measure, which had to be between 0.9 and 1.0. The features with the highest loading on the factors were selected.

## Results

Nineteen patients were included in the period between July 2018 and March 2020. All patients completed chemoradiotherapy, and all but one underwent surgery after an average of 14.1 ± 6.6 weeks (one cCR patient in watch-and-wait). All but one patient completed all six imaging studies: in one patient the final [^18^F]FDG PET/CT was not performed due to logistical problems. Sixteen men and three women were included in this study with a median age of 63.1 (56.3–67.0) years old. The median follow-up time was 11.6 (9.0–17.1) months. No recurrent disease was found. One patient had a cCR without regrowth during follow-up, 4 patients had a pTRG1, 9 pTRG3, 4 pTRG4, and 1 pTRG5. On the basis of the pTRG, five patients (26.3%) were good responders, and 14 (73.7%) were poor responders. There were no significant differences at baseline between groups regarding age, sex, cT stage, cN stage, EMVI, and tumor differentiation, as summarized in Table 1.

**Table 1:** Patient and tumor characteristics at baseline

		Good response (*n* = 5)	Poor response (*n* = 14)	*P* value
Age (years)	Mean ± SD	63.6 ± 11.08	62.9 ± 6.12	0.273
Sex	Male	4	12	0.770
	Female	1	2	
cT	2	1	0	0.363
	3	3	10	
	4	1	4	
cN	0	1	2	1.00
	1	0	1	
	2	4	11	
EMVI	Yes	0	3	0.565
	No	5	11	
	Missing	0	0	
Differentiation (biopsy)	Well/moderate	3	13	0.071
	Poor	1	0	
	Missing	1	1	

Table shows difference between the good and poor response groups at baseline with corresponding *P* value.

cT, clinical tumor stage on routine staging MRI; cN, clinical nodal stage on routine staging MRI; EMVI, extramural vascular invasion.

### Quantitative features

A total of 57 quantitative features were extracted. Redundancy filtering and factor analysis of the radiomic feature sets were performed and Kaiser-Meier-Olkin (**KMO**) measures were excellent (>0.96). The features corresponding best with the two factors per sequence and timepoint were included in the analysis.

Using binary logistic regression analysis with a predefined cutoff value of *P* ≤ 0.2, 13 features were found to be promising predictors of response. At baseline imaging, three MRI and two PET/CT features were found to be promising. At early response evaluation, no promising features were found; however, two MRI and two PET/CT early response evaluations to baseline response index features were found to be promising. At the end-of-treatment presurgical evaluation, three MRI and one PET/CT feature were found to be promising, but no response index features were promising.

**Fig. 1:**
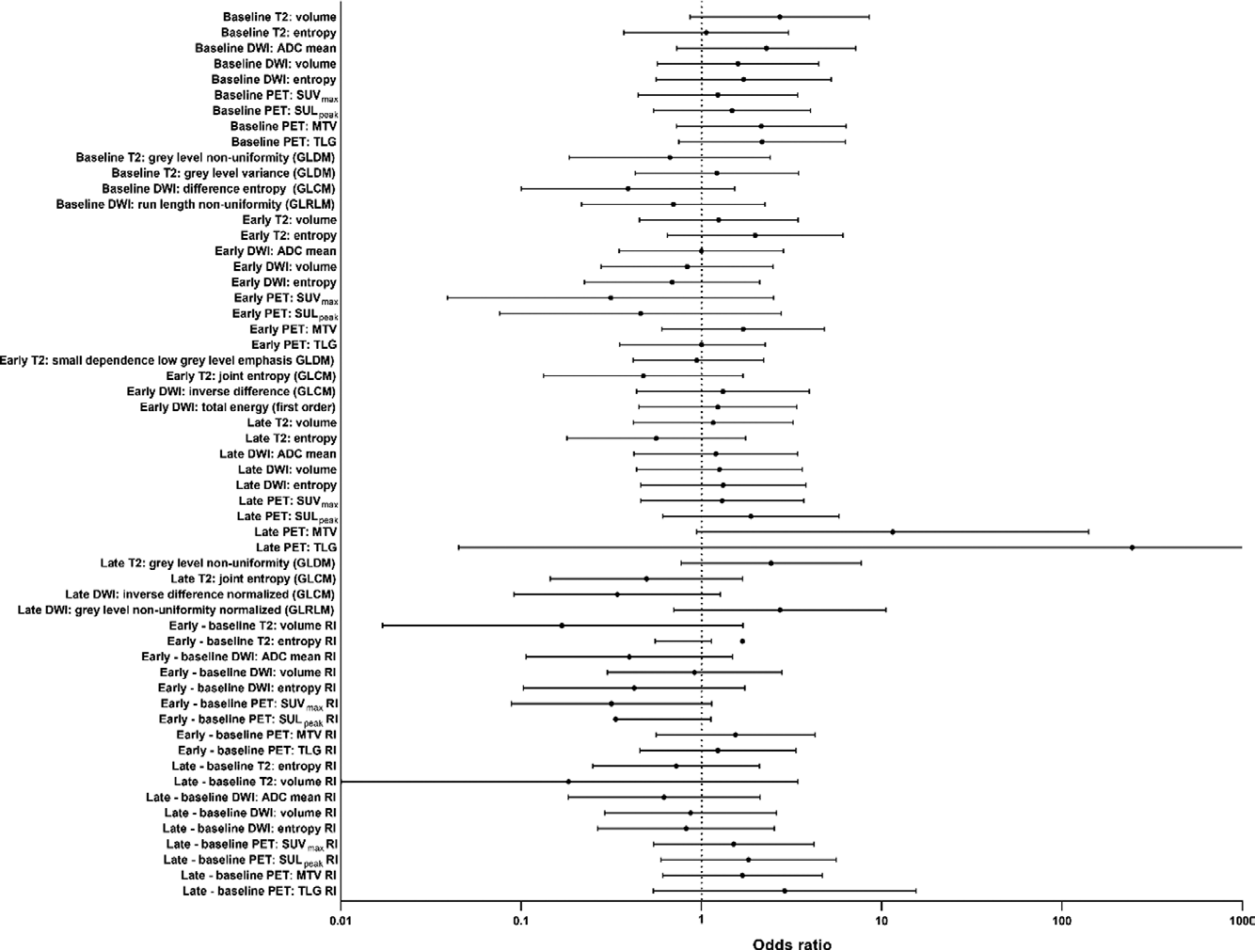
Forrest plot of investigated features. Figure shows odds ratio for TRG1-2 with 95% confidence intervals from binary logistical regression analyses on logarithmic scale (x-axis). ADC mean, mean apparent diffusion coefficient; DWI entropy, tumor entropy on diffusion-weighted imaging series; DWI volume, tumor volume on diffusion-weighted imaging series; MTV, metabolic tumor volume; SUL_peak_, peak standardized uptake value corrected for lean body mass; SUV_max_, maximum standardized uptake value; T2 entropy, tumor entropy on T2 series; T2 volume, tumor volume on T2 series; TLG, total lesion glycolysis.

**Fig. 2:**
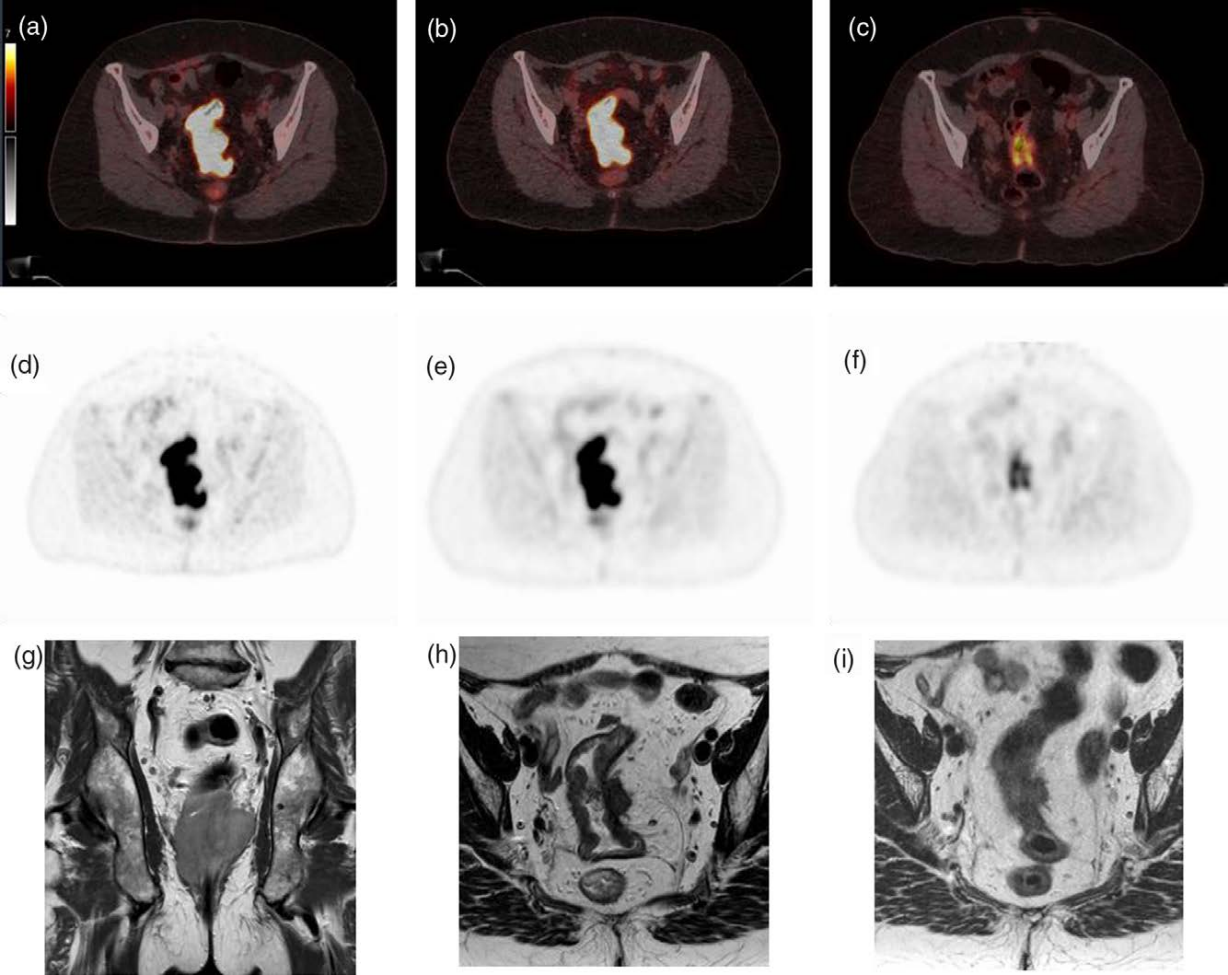
[^18^F]FDG PET/CT and T2 weighted MRI images of a poor responder before, during, and after neoadjuvant chemoradiotherapy. A fifty-eight-year-old woman with cT4aN2M0 rectal cancer had a partial response to chemoradiotherapy to a yiT3N1M0. Pathological examination showed a ypT3N0M0 tumor and pTRG of 4. SUV_max_ was 17.8 at baseline, 17.8 at interim assessment, and 6.5 at reevaluation. Figure shows [^18^F]FDG PET/CT fusion (a–c) and PET-only (d–f) images as well as T2 weighted MRI (g–i) images before (a, d, g), during (b, e, h) and after (c, f, i) neoadjuvant chemoradiotherapy. CT, computed tomography; pTRG, pathological tumor regression grade; SUV_max_, maximum standardized uptake value.

**Fig. 3:**
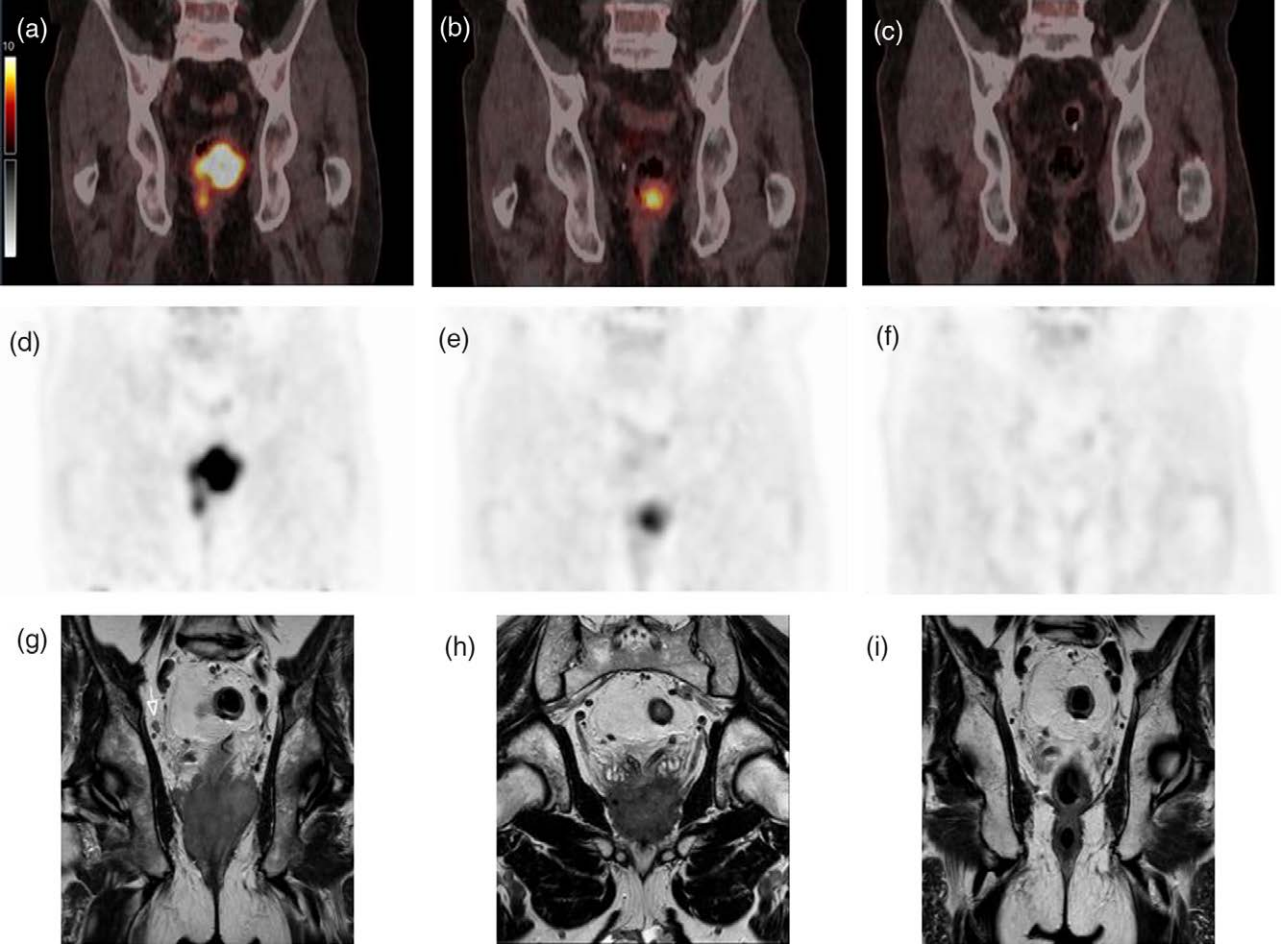
[^18^F]FDG PET/CT and T2 weighted MRI images before, during, and after neoadjuvant therapy of a patient with clinical complete response. A sixty-two-year-old man with cT4bN2M0 rectal cancer had a good response to a yiT1-2N0M0 which further regressed to a yiT0N0M0 6 months after chemoradiotherapy, and is currently still followed in the watch-and-wait after 12 months of recurrence-free follow-up. SUV_max_ was 18.1 at baseline, 10.4 at interim assessment, and too low to measure at reevaluation. Figure shows [^18^F]FDG PET/CT fusion (a–c) and PET-only (d–f) images as well as T2 weighted MRI (g–i) images before (a, d, g), during (b, e, h) and after (c, f, i) neoadjuvant chemoradiotherapy. CT, computed tomography; SUV_max_, maximum standardized uptake value.

These results are shown in more detail in the forest plot in Fig. 1, which displays all features with their respective odds ratios and confidence interval. It shows numerous features to have preferable odds ratios; however, only 13 have a *P* ≤ 0.2. Detailed results from binary logistic regression analysis are displayed in Table 2. Figures 2 and 3 present examples of good and poor responders on sequential multimodality imaging.

**Table 2:** Binary logistical regression analysis of MRI and PET/computed tomography features for prediction of response

				95% confidence interval		
Feature	Regression coefficient	Odds ratio	Standard error	Lower limit	Upper limit	*P* value
Baseline T2: tumor volume	0.999	2.716	0.584	0.864	8.537	0.087
Baseline T2: entropy	0.06	1.062	0.536	0.371	3.037	0.911
Baseline DWI: mean ADC	0.827	2.287	0.583	0.730	7.168	0.156
Baseline DWI: tumor volume	0.464	1.591	0.525	0.568	4.456	0.377
Baseline DWI: entropy	0.537	1.711	0.570	0.559	5.231	0.347
Baseline T2: gray level nonuniformity (GLDM)	−0.405	0.667	0.655	0.185	2.408	0.536
Baseline T2: gray level variance (GLDM)	0.195	1.216	0.531	0.429	3.445	0.713
Baseline DWI: difference entropy (GLCM)	−0.940	0.391	0.695	0.100	1.527	0.177
Baseline DWI: run length nonuniformity (GLRLM)	−0.360	0.697	0.598	0.216	2.250	0.546
Baseline PET: SUV_max_	0.210	1.233	0.520	0.445	3.418	0.687
Baseline PET: SUL_peak_	0.388	1.475	0.511	0.541	4.016	0.447
Baseline PET: MTV	0.764	2.147	0.552	0.728	6.337	0.166
Baseline PET: TLG	0.773	2.166	0.543	0.748	6.276	0.154
Early T2: tumor volume	0.220	1.246	0.517	0.452	3.435	0.670
Early T2: entropy	0.686	1.986	0.571	0.648	6.084	0.230
Early DWI: ADC mean	−0.003	0.997	0.536	0.349	2.848	0.995
Early DWI: tumor volume	−0.184	0.832	0.561	0.277	2.497	0.743
Early DWI: entropy	−0.376	0.687	0.571	0.224	2.104	0.511
Early T2: small dependence low gray level emphasis (GLDM)	−0.061	0.941	0.414	0.418	2.120	0.883
Early T2: joint entropy (GLCM)	−0.745	0.475	0.651	0.133	1.699	0.252
Early DWI: inverse difference (GLCM)	0.273	1.314	0.563	0.436	3.957	0.628
Early DWI: total energy (first order)	0.208	1.231	0.515	0.449	3.376	0.686
Early PET: SUV_max_	−1.159	0.314	1.061	0.039	2.508	0.274
Early PET: SUL_peak_	−0.778	0.459	0.916	0.076	2.764	0.396
Early PET: MTV	0.532	1.702	0.529	0.603	4.803	0.315
Early PET: TLG	0.001	1.001	0.535	0.351	2.856	0.999
Late T2: tumor volume	0.150	1.161	0.520	0.419	3.221	0.774
Late T2: entropy	−0.579	0.560	0.583	0.179	1.755	0.320
Late DWI: ADC mean	0.181	1.199	0.533	0.422	3.407	0.734
Late DWI: tumor volume	0.228	1.256	0.540	0.436	3.616	0.673
Late DWI: entropy	0.276	1.318	0.539	0.459	3.788	0.608
Late T2: gray level nonuniformity (GLDM)	0.889	2.432	0.587	0.770	7.687	0.130
Late T2: joint entropy (GLCM)	−0.703	0.495	0.626	0.145	1.688	0.261
Late DWI: inverse difference normalized (GLCM)	−1.077	0.341	0.672	0.091	1.271	0.109
Late DWI: gray level nonuniformity normalized (GLRLM)	1.001	2.722	0.692	0.702	10.558	0.148
Late PET: SUV_max_	0.263	1.301	0.531	0.459	3.686	0.621
Late PET: SUL_peak_	0.631	1.880	0.575	0.610	5.800	0.272
Late PET: MTV	2.441	11.480	1.278	0.937	140.578	0.056
Late PET: TLG	5.499	244.488	4.385	0.045	1, 320, 336.926	0.210
Early – baseline T2: tumor volume RI	−1.781	0.168	1.179	0.017	1.699	0.131
Early – baseline T2: entropy RI	0.522	1.685	0.568	0.553	5.134	0.359
Early – baseline DWI: ADC mean RI	−0.923	0.397	0.673	0.106	1.486	0.170
Early – baseline DWI: tumor volume RI	−0.089	0.915	0.569	0.300	2.789	0.875
Early – baseline DWI: entropy RI	−0.860	0.423	0.721	0.103	1.738	0.233
Early – baseline PET: SUV_max_ RI	−1.150	0.316	0.653	0.088	1.139	0.078
Early – baseline PET: SUL_peak_ RI	−1.096	0.334	0.621	0.099	1.129	0.078
Early – baseline PET: MTV RI	0.434	1.543	0.518	0.559	4.259	0.402
Early – baseline PET: TLG RI	0.210	1.233	0.509	0.455	3.347	0.680
Late – baseline T2: tumor volume RI	−1.700	0.183	1.496	0.010	3.427	0.256
Late – baseline T2: entropy RI	−0.325	0.723	0.544	0.249	2.098	0.550
Late – baseline DWI: ADC mean RI	−0.480	0.619	0.626	0.182	2.110	0.443
Late – baseline DWI: tumor volume RI	−0.141	0.869	0.560	0.290	2.603	0.801
Late – baseline DWI: entropy RI	−0.198	0.821	0.575	0.266	2.533	0.731
Late – baseline PET: SUV_max_ RI	0.410	1.507	0.522	0.541	4.197	0.432
Late – baseline PET: SUL_peak_ RI	0.600	1.823	0.571	0.595	5.582	0.293
Late – baseline PET: MTV RI	0.523	1.687	0.520	0.609	4.676	0.315
Late – baseline PET: TLG RI	1.062	2.892	0.856	0.540	15.482	0.215

Table shows regression coefficient, odds ratios with confidence intervals, and *P* values.

SUV_max_, maximum standardized uptake value; SUL_peak_, peak standardized uptake value corrected for lean body mass; MTV, metabolic tumor volume; TLG, total lesion glycolysis; T2 volume, tumor volume on T2 series; T2 entropy, tumor entropy on T2 series; DWI volume, tumor volume on diffusion-weighted imaging series; ADC mean, mean apparent diffusion coefficient; DWI entropy, tumor entropy on diffusion-weighted imaging series; GLCM: gray level cooccurrence matrix, GLDM: gray level dependence matrix, GLRLM: gray level run length matrix, RI, response index (change over time).

## Discussion

Results from this pilot study indicate that 13 out of 57 features are promising predictors of response, with baseline and early change showing the most clinically relevant features. As deduced from these results, end-of-treatment presurgical evaluation digital PET/CT was least probable to provide predictive (and clinically relevant) features. As far as we know, this is the first prospective study in LARC patients investigating the predictive value of multiparametric MRI and digital [^18^F]FDG PET/CT, at three set time points during neoadjuvant chemoradiation.

The results from this study confirm the feasibility of response prediction using digital [^18^F]FDG PET/CT and multiparametric MRI. These results are in line with previous reports from various small trials demonstrating the predictive value of various T2- and DW MRI and [^18^F]FDG PET/CT features, which have up until now not resulted in clinically usable prediction models [14,17]. In contrast to our results, a recent study in 19 LARC patients found only baseline MTV and no early response evaluation features (2 weeks into nCRT) to be possible predictors of response [31]. In our study we also found baseline MTV to be a promising feature; however, we also found four other baseline features (three MRI, one PET) and four early response evaluation response indices features (two MRI, two PET). Interestingly, they found more predicting features at end-of-treatment presurgical evaluation [^18^F]FDG PET/CT (SUV_max_, SUV_peak_, MTV, SUL_peak_, TLG), whereas our study only found MTV to be a promising feature (note that the exact timing of the late evaluation [^18^F]FDG PET/CT in their study is unclear). As a next step towards clinical implementation, Schurink *et al.* developed prediction models including features from MRI and [^18^F]FDG PET/CT that were also used in the current study. The first study found an area under the curve (AUC) of 0.83 for response prediction at baseline using MRI-derived T-stage, T2 entropy, and T2 volume [19]. The second study found an AUC of 0.83 using clinical (T-stage, N-stage, age, sex, interval between nCRT and end-of-treatment presurgical evaluation) and baseline features (T2 entropy, ADC entropy, and SUV_mean_) [20]. Interestingly, models including radiomic features did not outperform the simpler model [20]. Moreover, radiomic analysis of PET/CT images (AUC 0.78) did outperform simpler features (SUV_mean_, TLG, and mean Hounsfield unit, AUC 0.50) [20]; however, PET/CT radiomic analyses were performed on the CT-only images, thus questioning the added value of PET. In comparison to our study, in which MRI-based radiomic features were analyzed, we found four out of 12 radiomic features to be promising predictors of response (one baseline and three end-of-treatment presurgical evaluation features). Unfortunately, no AUC values were available due to the limited number of patients. Interestingly, the end-of-treatment presurgical evaluation [^18^F]FDG PET/CT was the least promising in this dataset. This might be due to the occurrence of radiation-induced proctitis interfering with end-of-treatment presurgical evaluation PET/CT, since inflammation results in increased uptake of [^18^F]FDG and is not present at early response evaluation yet.

Although accurate response prediction is currently challenging, the significant number of unidentified complete responders who undergo surgical resection stresses the importance of accurate response assessment and prediction. Following our results, a future trial should include multiparametric MRI at all three time points, and [^18^F]FDG PET/CT at baseline and early response evaluation. Furthermore, the sample size should be sufficient to define cutoff values and develop accurate prediction models. While this study focused primarily on predicting response using imaging modalities, the (combined) use of readily available predictive features such as metabolomics and analysis of biopsy material, and the integration of these in prediction models might further increase the accuracy of response prediction.

As inherent to any pilot study, this trial has various limitations. Due to the inclusion of only 19 patients and analysis of 57 features, no definite conclusions can be drawn from the data but only suggestions can be given toward design of future clinical trials. Due to the multicentric execution of this study various MRI scanners, with varying field strength, from various vendors, and with varying scanning protocols were used. This introduces heterogeneity in the quantitative MRI features. Nevertheless, this reflects the clinical routine as the acquisition of a larger dataset of LARC patients requires inclusion from multiple hospitals. Preferably quantitative parameters would be compared from the various MRI scanners, protocols, and field strengths; however, such a dataset is currently unavailable. A previous study by Mes *et al.*, however, found minimal influence of varying signal intensities from various MRI scanners on the outcome of radiomics analysis, thus suggesting the influence of this heterogeneity to be limited [high concordance (mean 0.82 ± 0.19) for 89 radiomics features before and after gray level normalization] [32]. Most recently, Schurink *et al.* investigated the influence of multiple MRI vendors and acquisition protocols on radiomic analysis in 649 rectal cancer patients [33]. They found significant differences in image features between nine centers, with more differences found in ADC/DWI imaging compared to T2-weighted MRI. Last, inter-observer variability has been introduced as the TRG was determined by various local pathologists; however, as the data were divided into only two groups, the influence of this was deemed minimal. Future studies should take these issues into account, and either further investigate the possible influence of various scanner types and acquisition protocols, perform the study on one MRI scanner within the same institute, or develop methods to harmonize the data. Also, a future study should consider the possible shift toward the use of more short-course radiotherapy combined with systemic chemotherapy following results from the RAPIDO trial, as opposed to CRT as described by current guidelines [3]. This issue is less relevant for pooling data from [^18^F]FDG PET/CT because data are (largely) harmonized by following the EANM guidelines and only one single PET/CT scanner was used in this study [22].

In conclusion, results from this study suggest that baseline, early response and end-of-treatment presurgical evaluation MRI and baseline and early response evaluation PET/CT features are promising to predict response to neoadjuvant therapy in rectal cancer patients. These results, in combination with the clinical need for improved treatment stratification, encourage further research into response prediction using [^18^F]FDG PET/CT and multiparametric MRI.

## Acknowledgements

The authors thank Lieveke Michielsens for her aid in patient selection during this trial. This study was financially supported by a European Research Council Advanced Grant (C.J.H. van de Velde, no. 323105), the Dutch Cancer Society (KWF) Bas Mulder Award (D.E.H., no. UL 2015-7966), and a Horizon2020 grant (A.L.V., no. 857894). F.V., S.F., W.N., F.v.V., L.G., H.P., and D.H. wrote the main manuscript text and performed the analysis. F.V., F.v.V., F.P., L.G., and D.H. were involved in the trial design. All other authors reviewed and agreed upon the manuscript. The study was conducted in accordance with the Declaration of Helsinki, and approved by the Institutional Review Board (or Ethics Committee) of Leiden Den Haag Delft. Written informed consent was obtained from all subjects (patients) in this study. Data is available upon reasonable request from the corresponding author.

### Conflicts of interest

There are no conflicts of interest.

## Supplementary Material


