# Supplementary material for: Baseline and early digital [18F]FDG PET/CT and multiparametric MRI contain promising features to predict response to neoadjuvant therapy in locally advanced rectal cancer patients: a pilot study
Source: Nucl Med Commun. 2023 May 3;44(7):613–21. doi: 10.1097/MNM.0000000000001703 (PMC10246883; doi:10.1097/MNM.0000000000001703)
Supplement: Supplementary file 1 [file nmc-44-613-s001.pdf]

Supplemental Table 1. Specifications of MRI scanners. Table shows specifications of various MRI scanners used in this study.

**Vendor**	**Field strength (Tesla)**	**Number of scans**	**Acquisition voxel size (mm)**
Philips Ingenia	1.5	6	0.62 x 0.81
Philips Ingenia	1.5	2	1.49 x 2.00
Philips Ingenia	1.5	15	0.49 x 0.64
Philips Ingenia	3	1	0.94 x 1.25
Philips Ingenia	3	1	0.49 x 0.62
Philips Ingenia	3	7	0.49 x 0.62
Philips Ingenia Elition X	3	9	0.70 x 0.79
Philips Ingenia Elition X	3	1	0.70 x 0.53
Philips Ingenia Elition X	3	1	0.89 x 1.56
Siemens Avanto	1.5	1	0.69 x 0.80
Siemens Avanto	1.5	1	0.63 x 0.73
Siemens Avanto	1.5	1	0.59 x 0.78
Siemens Avanto	1.5	1	0.47 x 0.67
Siemens Avanto	1.5	1	0.51 x 0.72
Siemens Avanto	1.5	1	0.63 x 0.79
Siemens Aera	1.5	2	0.47 x 0.59
Siemens Aera	1.5	6	0.63 x 0.78
MRI sequences included T2 weighted sequences in sagittal, oblique axial and oblique coronal direction and an orthogonal diffusion weighted imaging (DWI) sequence (*b*-values 0, 200, and 800/1000, identical direction of T2 oblique axial). Oblique axial scans were perpendicular to the long axis of the rectal wall.			
